# Supplementary material for: Mitochondrial fission controls astrocyte morphogenesis and organization in the cortex
Source: J Cell Biol. 2025 Sep 3;224(10):e202410130. doi: 10.1083/jcb.202410130 (PMC12406776; doi:10.1083/jcb.202410130)
Supplement: SourceData F6 — is the source file for Fig. 6. [file jcb_202410130_sourcedataf6.pdf]

Source Data Figure 6

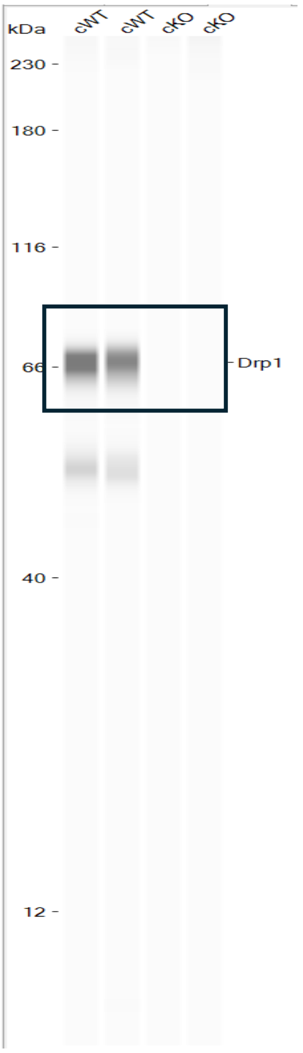

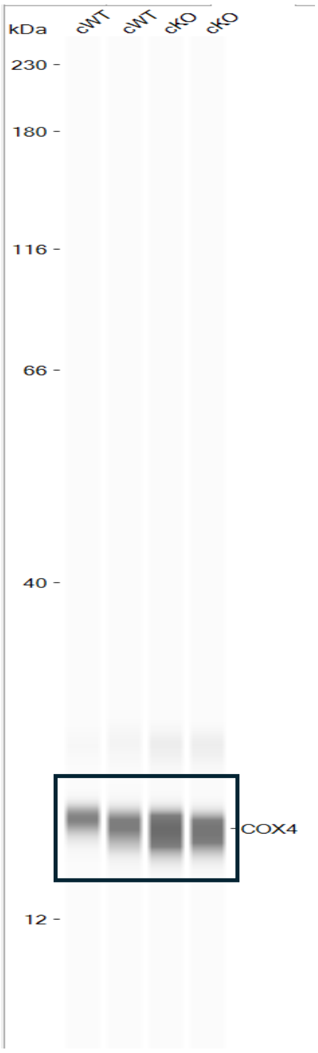

Source Data Figure 6 (Cont'd)

N of 1&2

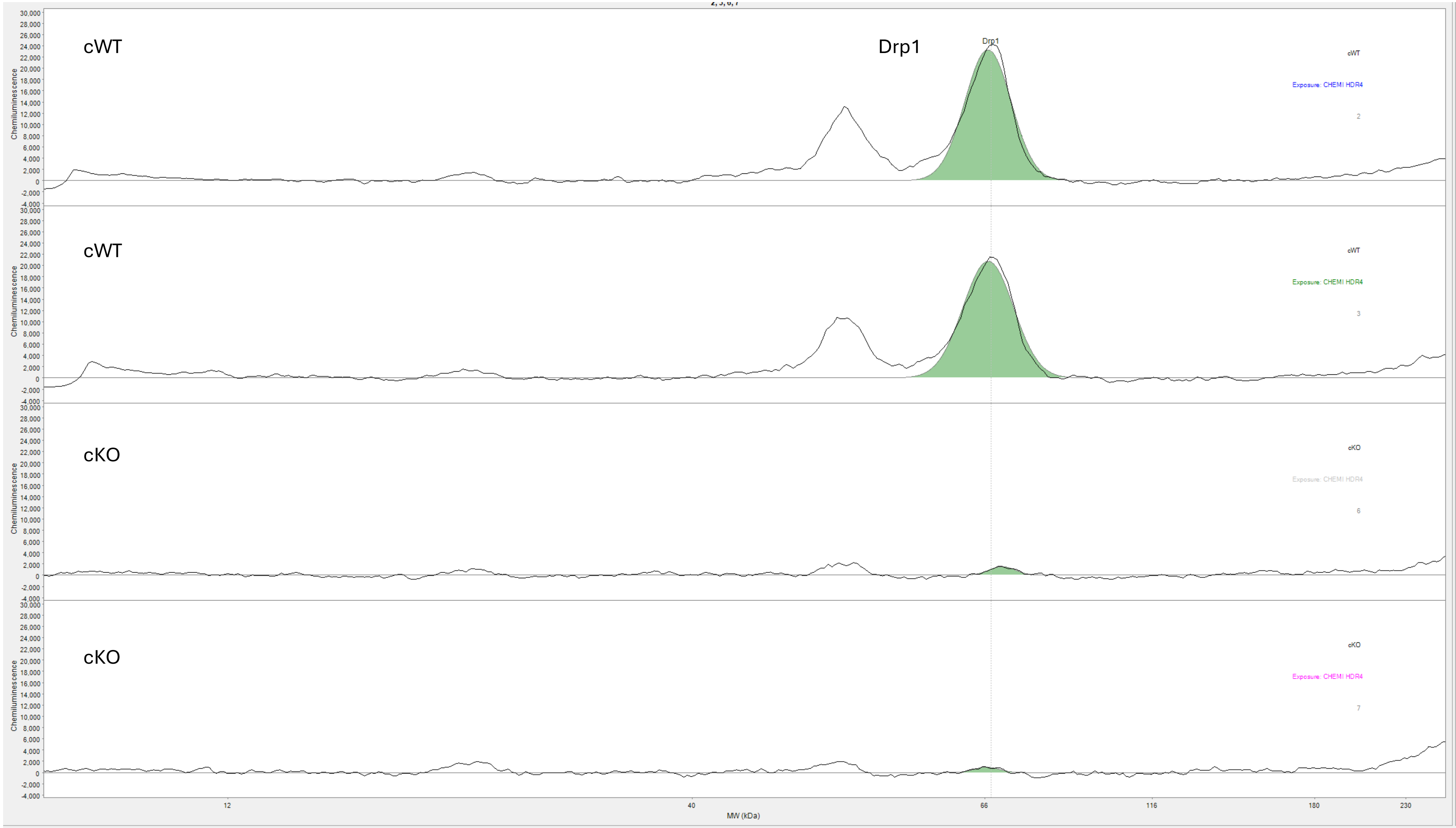

Peaks | Capillaries

Sample	Prim...	Seco...	Cap	Peak	Name	Positi...	MW (...	Height	Area	% Area	Width	S/N	Baseline	Channel
cWT	Rb ...	Seco...	2	1	Drp1	448	67	2332...	371475.3	100.0	15.0	14...	6258.2	CHEMI
cWT	Rb ...	Seco...	3	1	Drp1	447	67	2078...	361781.6	100.0	16.4	13...	6483.8	CHEMI
cKO	Rb ...	Seco...	6	1	Drp1	447	71	1538.4	13701.9	100.0	8.4	7.7	6106.0	CHEMI
cKO	Rb ...	Seco...	7	1	Drp1	448	66	978.2	8434.3	100.0	8.1	5.8	6856.0	CHEMI

Source Data Figure 6 (Cont'd)

N of 1&2

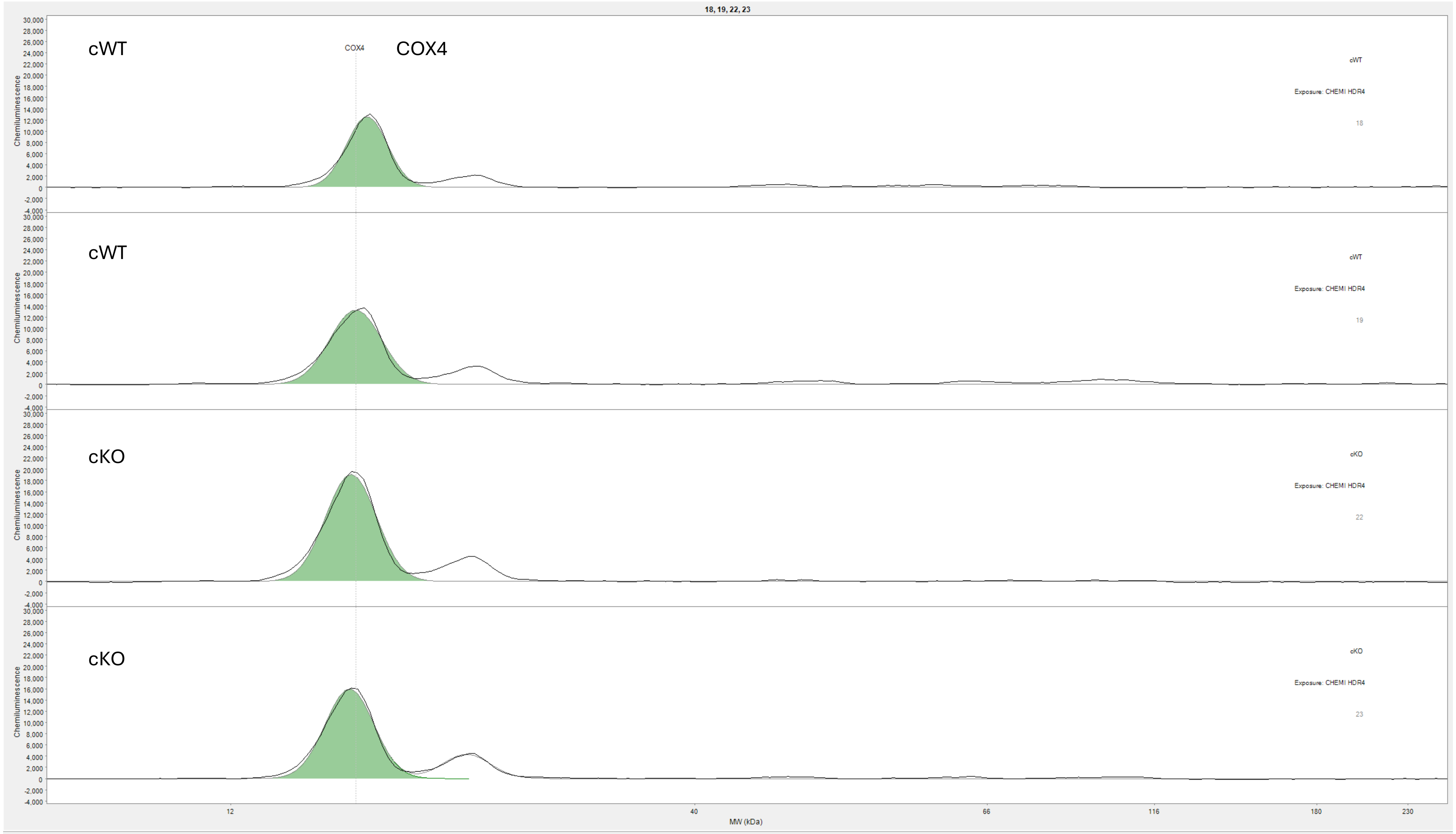

Peaks | Capillaries

Sample	Prim...	Seco...	Cap	Peak	Name	Positi...	MW (...	Height	Area	% Area	Width	S/N	Baseline	Channel
cWT	Rb ...	Seco...	18	1	COX4	283	20	1260...	117440.4	100.0	8.8	43...	395.2	CHEMI
cWT	Rb ...	Seco...	19	1	COX4	284	20	1326...	140215.4	100.0	9.9	38...	421.6	CHEMI
cKO	Rb ...	Seco...	22	1	COX4	284	19	1909...	196779.3	100.0	9.7	55...	488.3	CHEMI
cKO	Rb ...	Seco...	23	1	COX4	286	19	1598...	158477.7	100.0	9.3	64...	412.5	CHEMI

Source Data Figure 6 (Cont’d)

N of 3

Chemi raw image

Black and white raw image

Drp1 (75kDa)

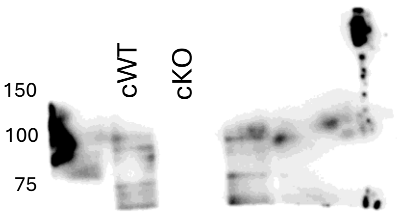

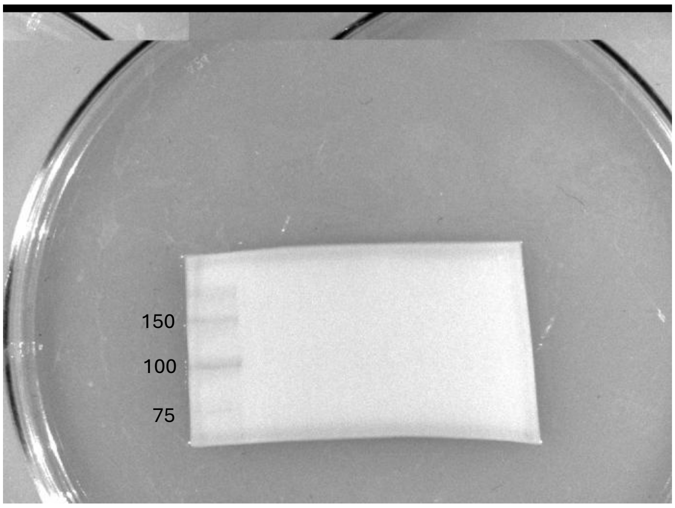

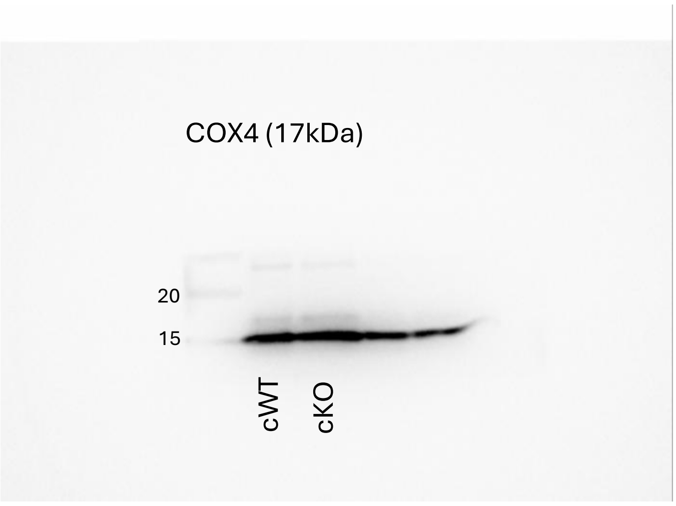

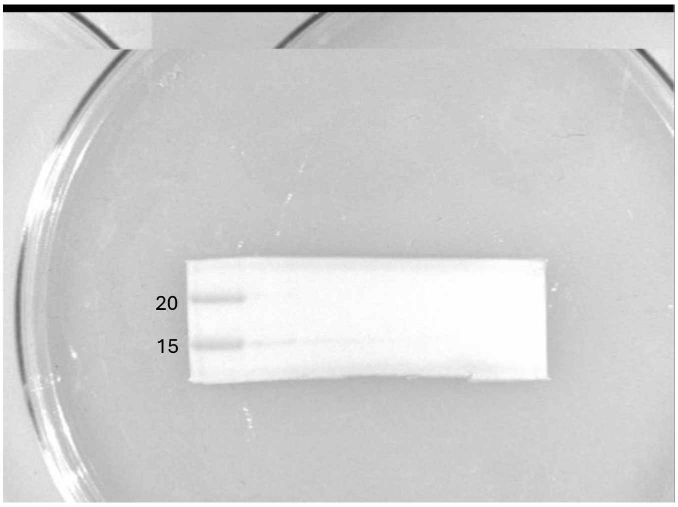

Lanes

1. cWT Astrocyte
2. cKO Astrocyte
3. cWT Flow through (not quantified)
4. cKO Flow through (not quantified)
