# Supplementary material for: Mitochondrial fission controls astrocyte morphogenesis and organization in the cortex
Source: J Cell Biol. 2025 Sep 3;224(10):e202410130. doi: 10.1083/jcb.202410130 (PMC12406776; doi:10.1083/jcb.202410130)
Supplement: SourceData F7 — is the source file for Fig. 7. [file jcb_202410130_sourcedataf7.pdf]

Source Data Figure 7

Panel I

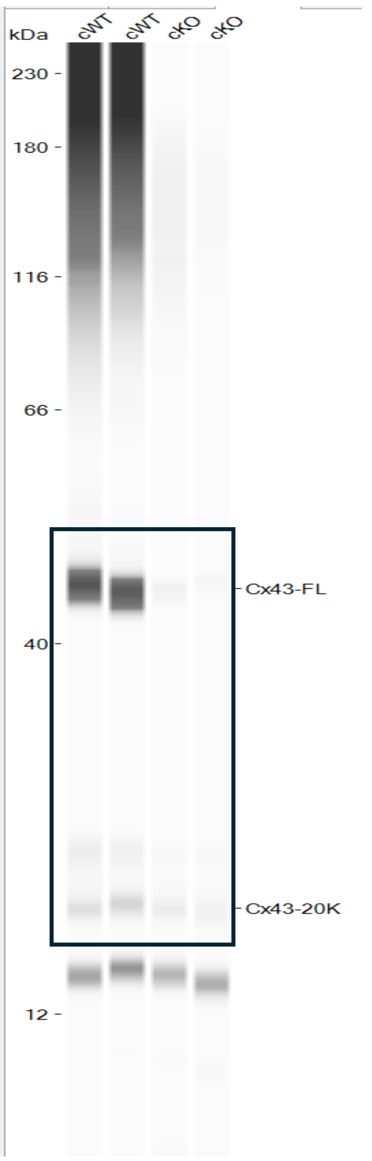

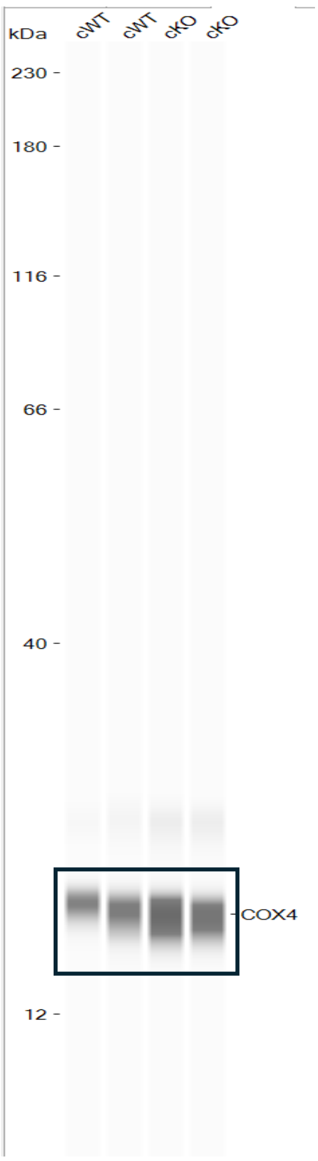

Source Data Figure 7 (Cont'd)

N of 1&2

Panel I

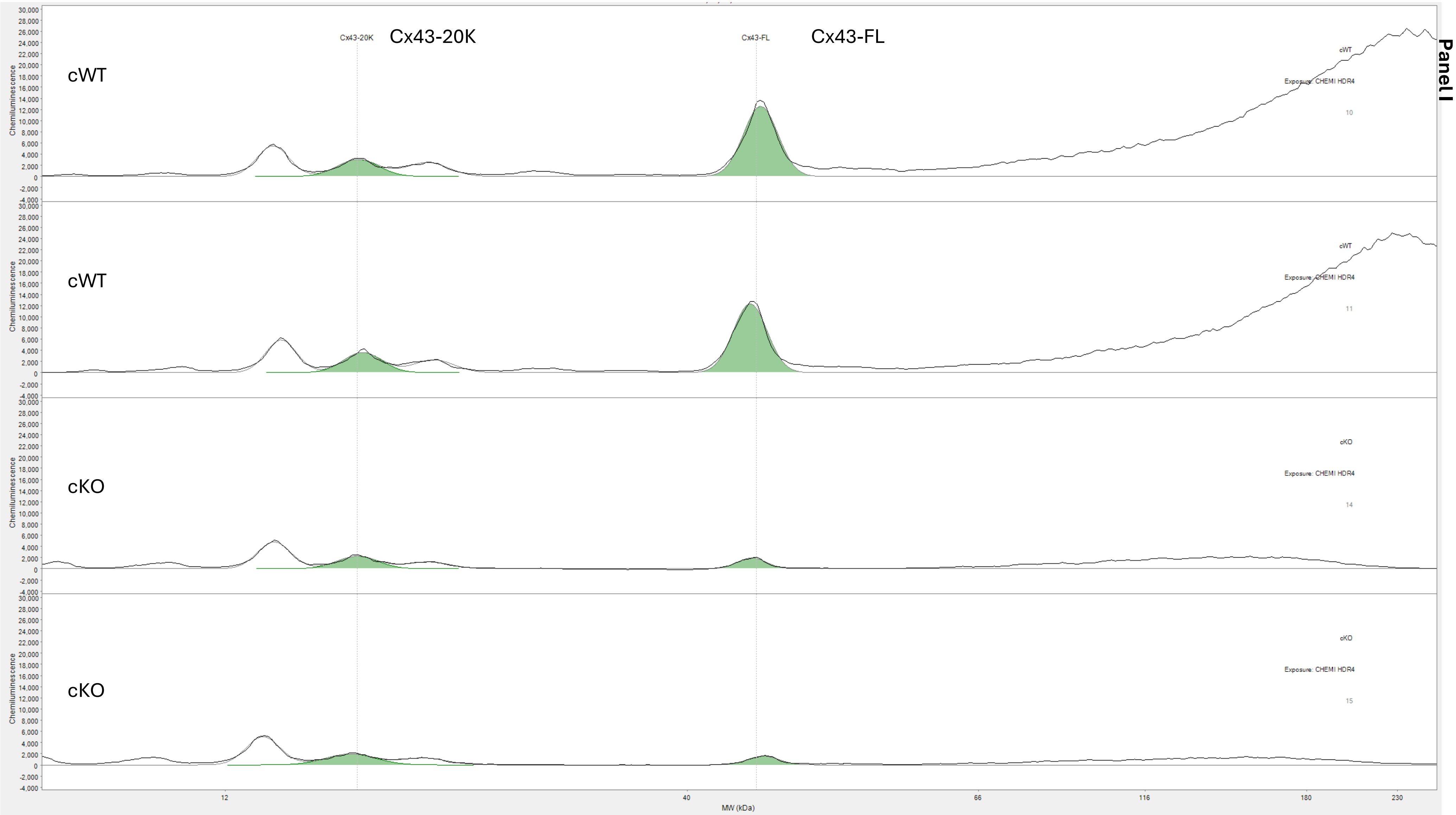

Peaks | Capillaries

Sample	Prim...	Seco...	Cap	Peak	Name	Positi...	MW (...	Height	Area	% Area	Width	S/N	Baseline	Channel
cWT	Rb ...	Seco...	10	2	Cx4...	271	20	3052.4	43648.2	22.6	13.4	28...	61.0	CHEMI
cWT	Rb ...	Seco...	10	4	Cx4...	382	47	1259...	149609.0	77.4	11.2	11...	57.8	CHEMI
cWT	Rb ...	Seco...	11	2	Cx4...	272	20	3624.7	50045.1	25.1	13.0	42...	42.6	CHEMI
cWT	Rb ...	Seco...	11	4	Cx4...	383	46	1234...	148991.2	74.9	11.3	14...	41.5	CHEMI
cKO	Rb ...	Seco...	14	2	Cx4...	272	20	2240.5	32611.4	62.2	13.7	23...	162.8	CHEMI
cKO	Rb ...	Seco...	14	4	Cx4...	382	46	1953.9	19822.9	37.8	9.5	20...	292.9	CHEMI
cKO	Rb ...	Seco...	15	2	Cx4...	272	20	1963.7	33207.4	66.7	15.9	28...	121.3	CHEMI
cKO	Rb ...	Seco...	15	4	Cx4...	381	47	1579.7	16570.1	33.3	9.9	23...	181.2	CHEMI

Source Data Figure 7

N of 1&2

Panel I

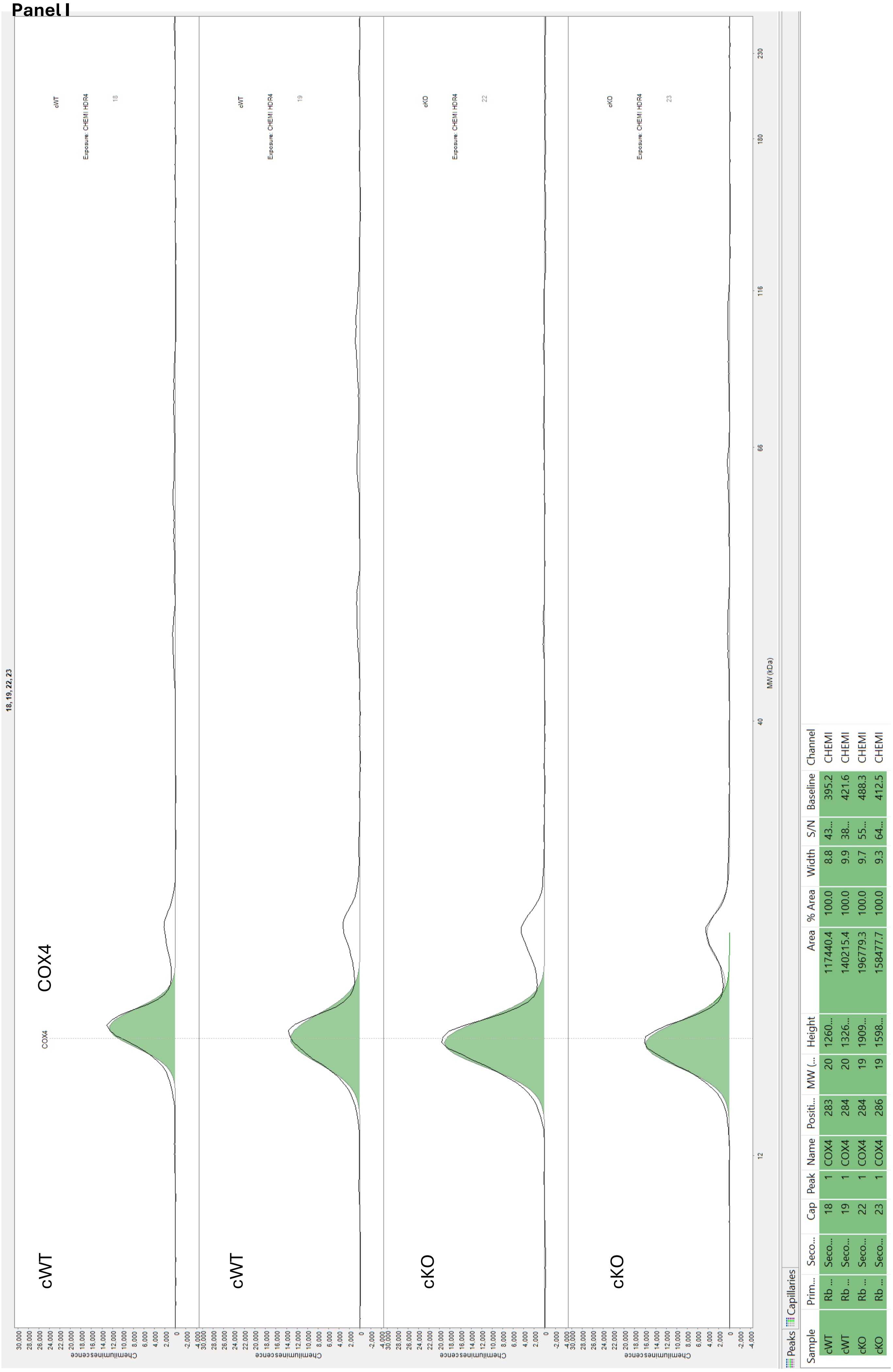

Sample	Prim...	Seco...	Cap	Peak	Name	Positi...	MW (...	Height	Area	% Area	Width	S/N	Baseline	Channel
cWT	Rb ...	Seco...	18	1	COX4	283	20	1260...	117440.4	100.0	8.8	43...	395.2	CHEMI
cWT	Rb ...	Seco...	19	1	COX4	284	20	1326...	140215.4	100.0	9.9	38...	421.6	CHEMI
cKO	Rb ...	Seco...	22	1	COX4	284	19	1909...	196779.3	100.0	9.7	55...	488.3	CHEMI
cKO	Rb ...	Seco...	23	1	COX4	286	19	1598...	158477.7	100.0	9.3	64...	412.5	CHEMI

Source Data Figure 7 (Cont’d)

N of 3

Panel I

Chemi raw image

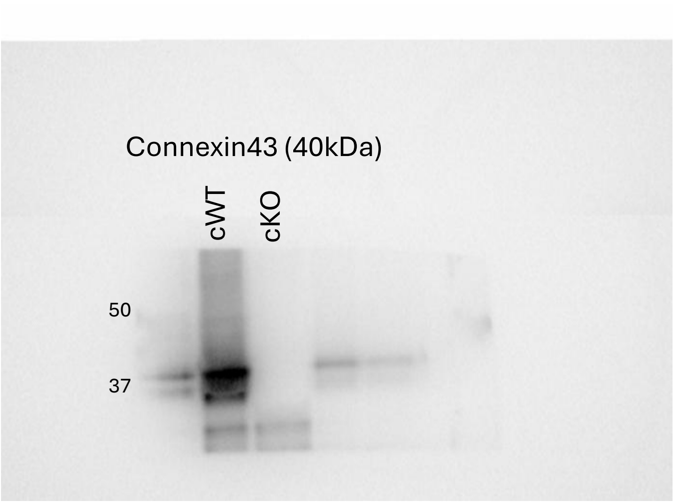

Black and white raw image

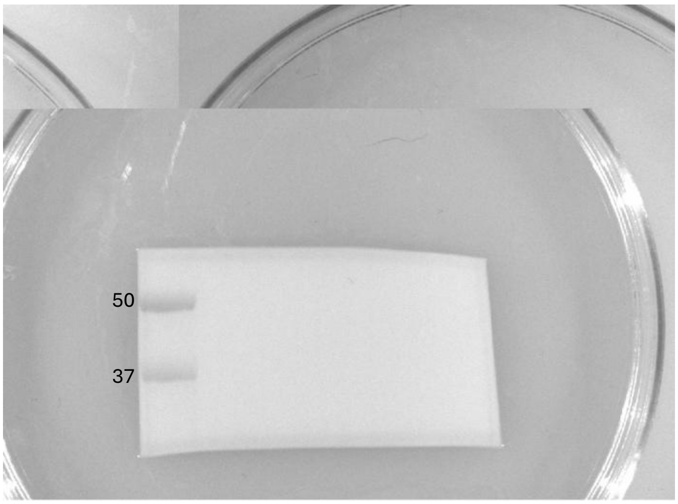

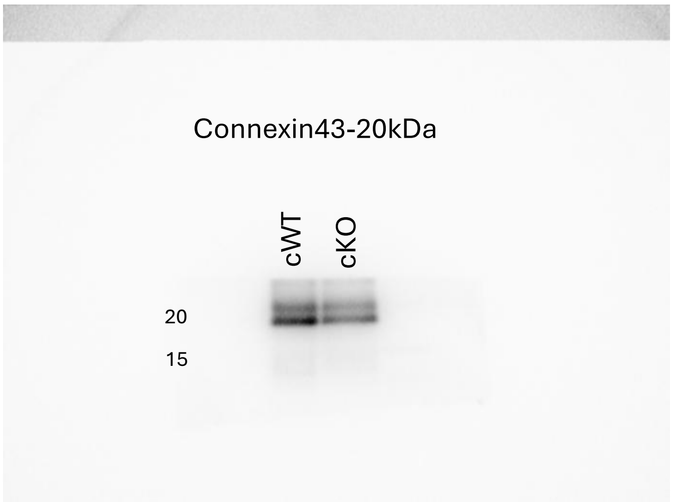

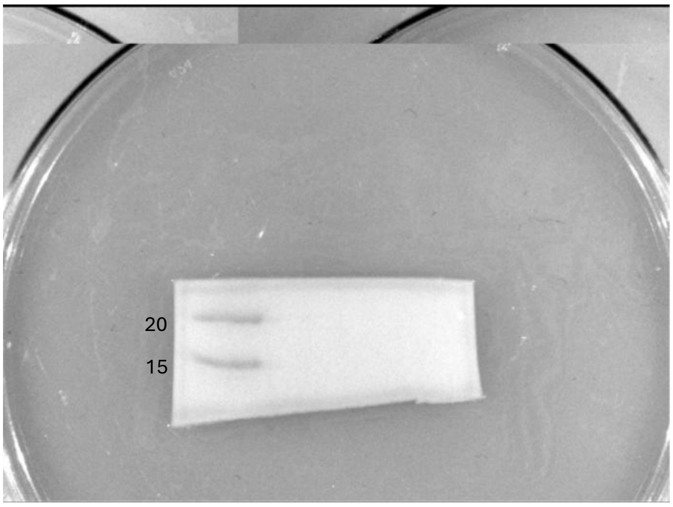

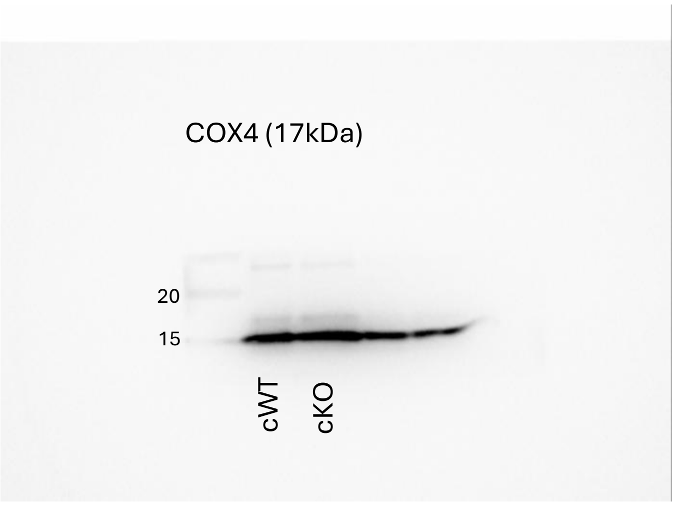

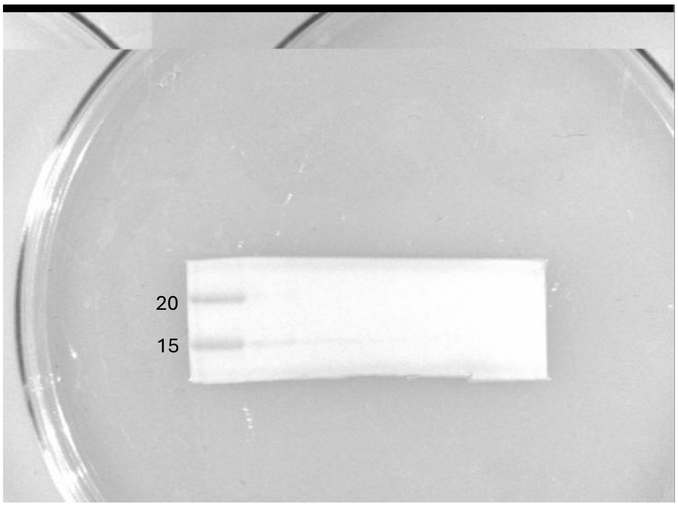

Lanes

1. cWT Astrocyte
2. cKO Astrocyte
3. cWT Flow through (not quantified)
4. cKO Flow through (not quantified)

Source Data Figure 7 (Cont'd)

N of 1&2

Panel L

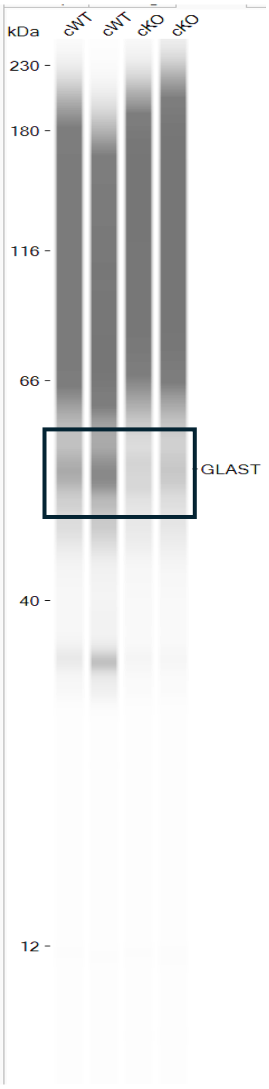

Panel N

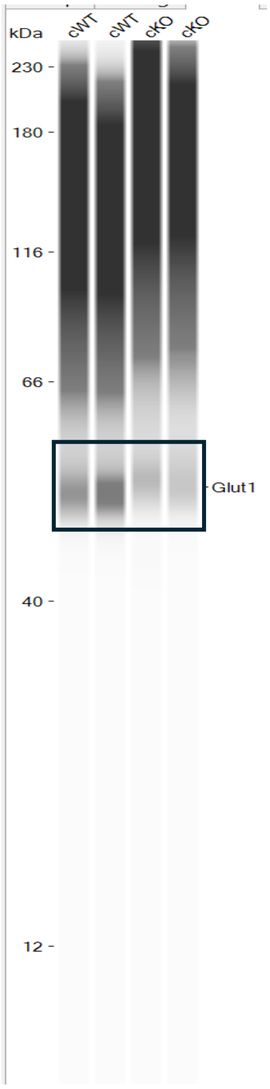

Panel P

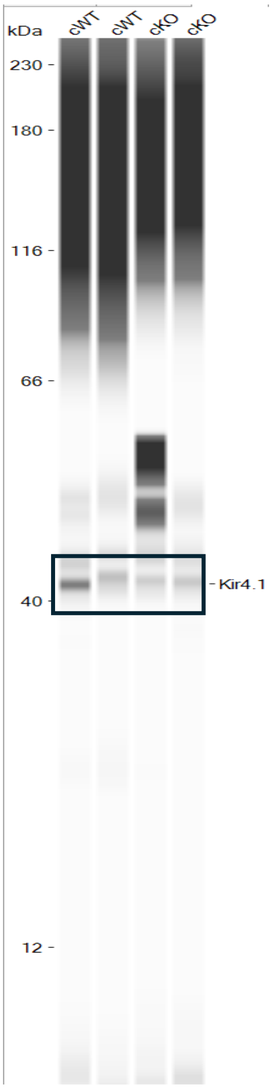

Panel L, N, P (loading control)

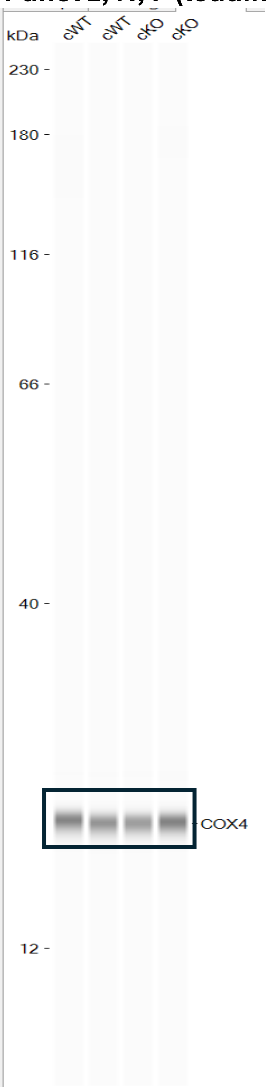

Source Data Figure 7 (Cont'd)

Panel L

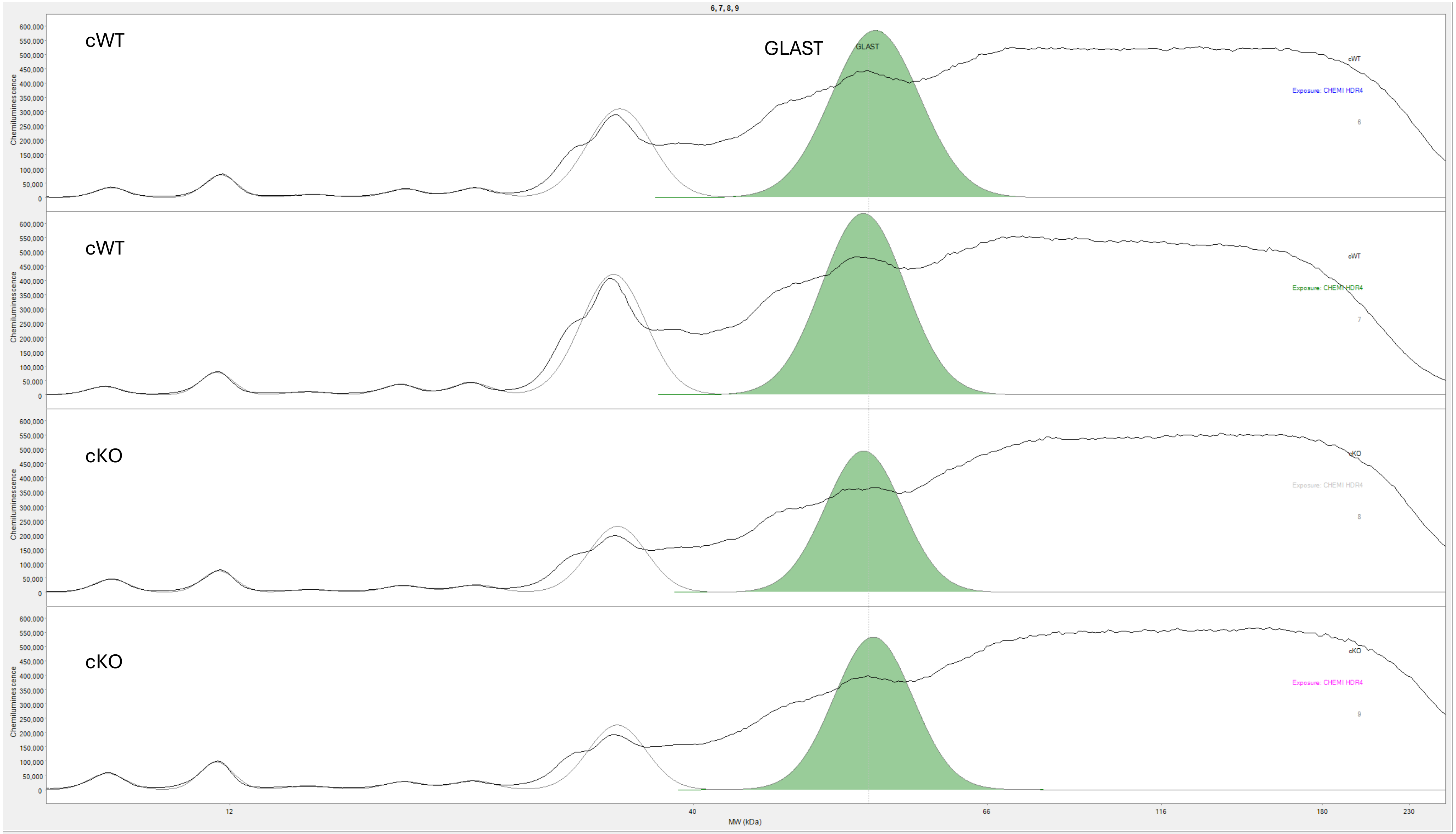

Peaks | Capillaries

Sample	Prim...	Seco...	Cap	Peak	Name	Positi...	MW (...	Height	Area	% Area	Width	S/N	Baseline	Channel
cWT	Rb ...	Seco...	6	7	GLA...	408	56	5844...	19641997.0	100.0	31.6	13...	1158.7	CHEMI
cWT	Rb ...	Seco...	7	7	GLA...	407	55	6352...	20150485.0	100.0	29.8	12...	1198.7	CHEMI
cKO	Rb ...	Seco...	8	7	GLA...	405	55	4947...	14470376.5	100.0	27.5	96...	969.0	CHEMI
cKO	Rb ...	Seco...	9	7	GLA...	407	56	5342...	16018686.7	100.0	28.2	26...	357.3	CHEMI

Source Data Figure 7 (Cont'd)

Panel N

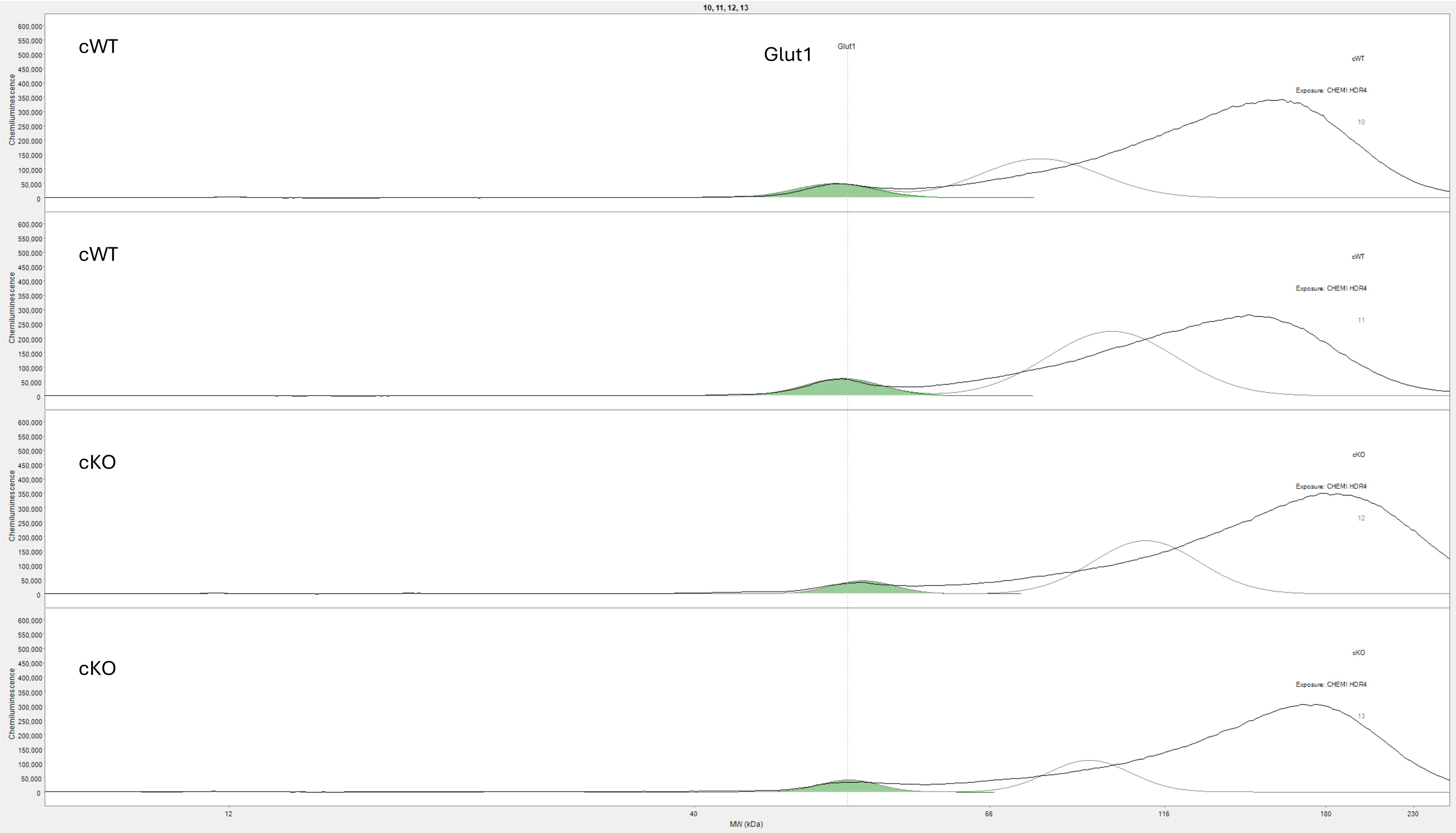

Peaks Capillaries

Sample	Prim...	Seco...	Cap	Peak	Name	Positi...	MW (kDa)	Height	Area	% Area	Width	S/N	Baseline	Channel
cWT	Rb ...	Seco...	10	4	Glut1	400	52	4933...	1523608.3	100.0	29.0	56...	3585.2	CHEMI
cWT	Rb ...	Seco...	11	4	Glut1	400	53	6081...	1779947.7	100.0	27.5	75...	3010.8	CHEMI
cKO	Rb ...	Seco...	12	4	Glut1	402	55	4597...	1078781.6	100.0	22.0	96...	2052.5	CHEMI
cKO	Rb ...	Seco...	13	4	Glut1	399	54	4227...	960852.6	100.0	21.4	66...	1895.6	CHEMI

Source Data Figure 7 (Cont'd)

Panel P

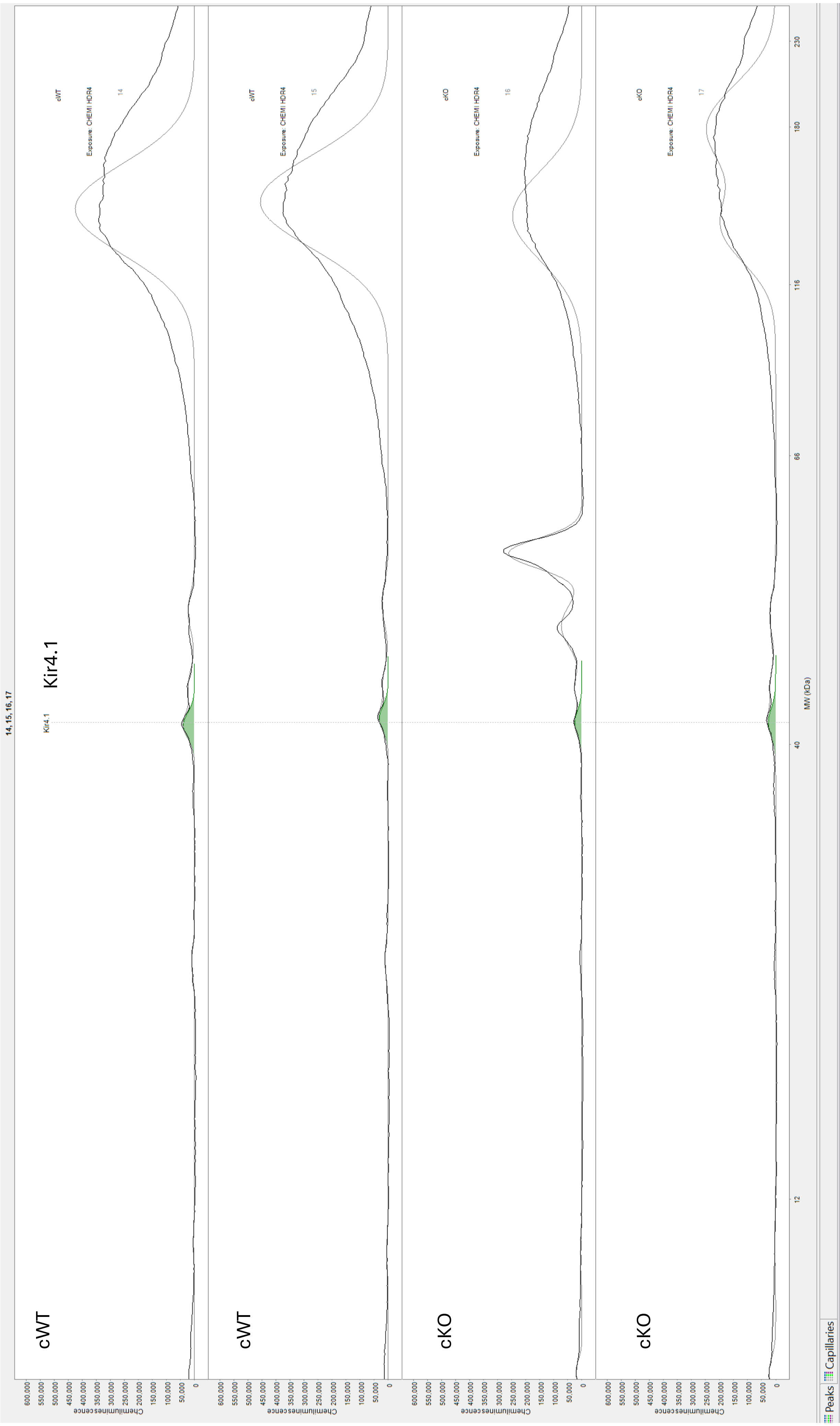

Sample	Prim...	Seco...	Cap	Peak	Name	Positi...	MW (kDa)	Height	Area	% Area	Width	S/N	Baseline	Channel
cWT	Rb ...	Seco...	14	3	Kir4.1	361	42	3994...	401884.5	100.0	9.5	35.2	55179.6	CHEMI
cWT	Rb ...	Seco...	15	3	Kir4.1	363	42	3159...	304620.1	100.0	9.1	33.3	54149.9	CHEMI
cKO	Rb ...	Seco...	16	3	Kir4.1	363	42	2608...	262170.7	100.0	9.4	37.3	57126.7	CHEMI
cKO	Rb ...	Seco...	17	3	Kir4.1	365	42	2622...	284034.0	100.0	10.2	38.9	52343.9	CHEMI

Source Data Figure 7 (Cont'd)

Panel L, N, P (loading control)

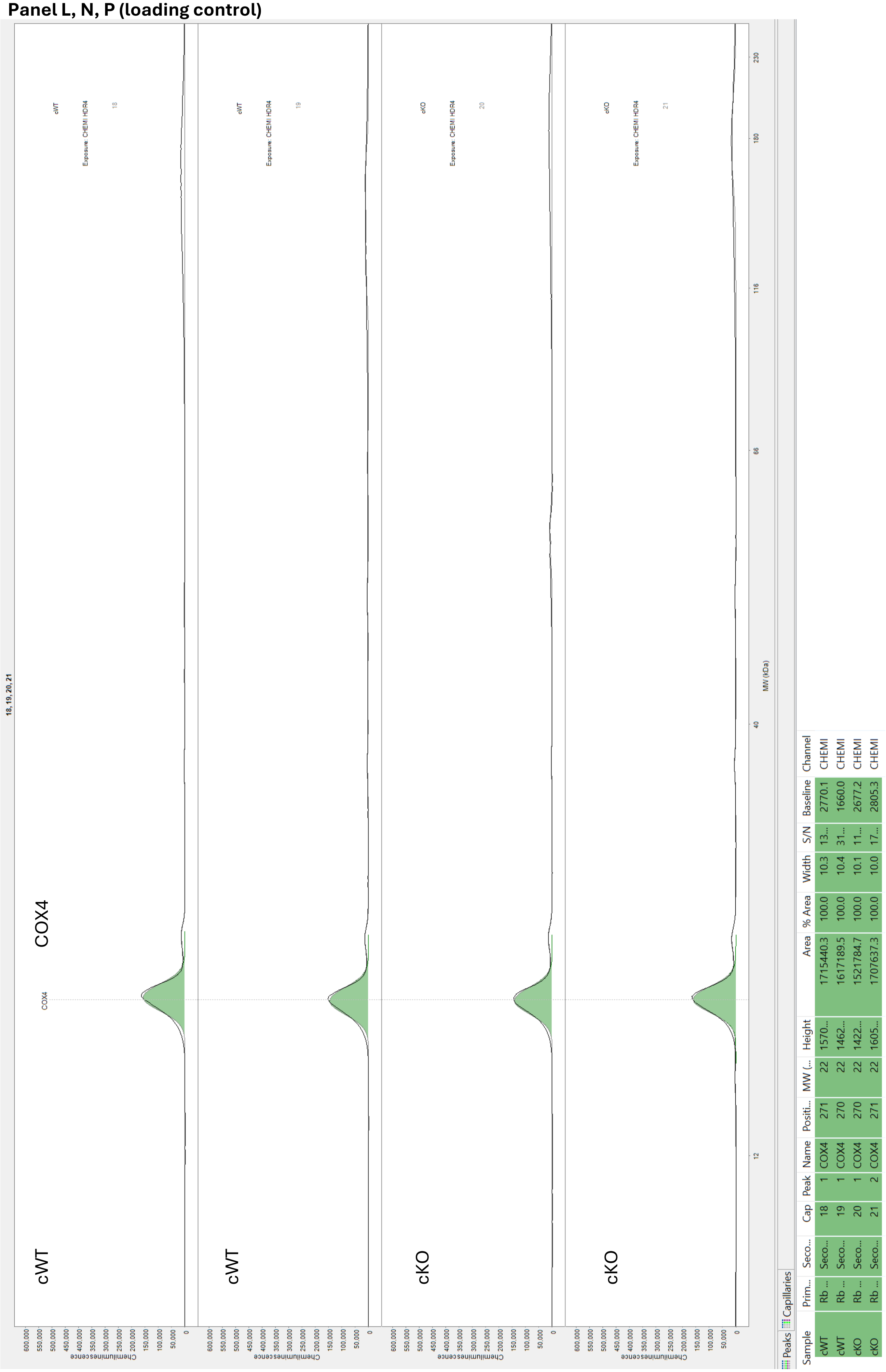

Source Data Figure 7 (Cont’d)

N of 3&4

Panel L

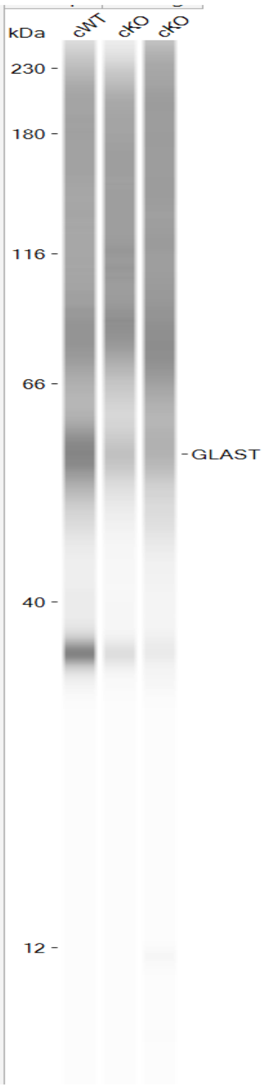

Panel N

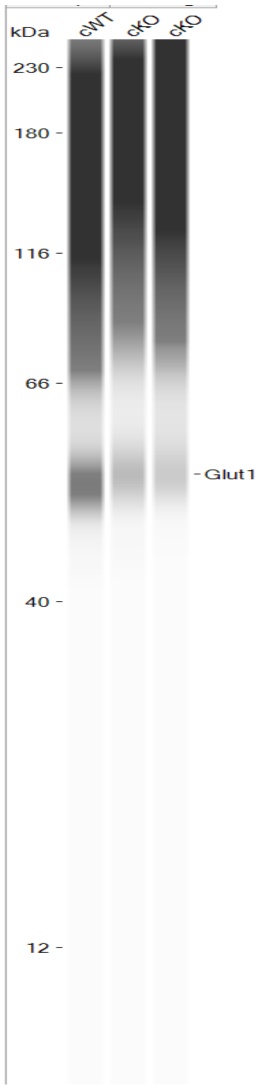

Panel P

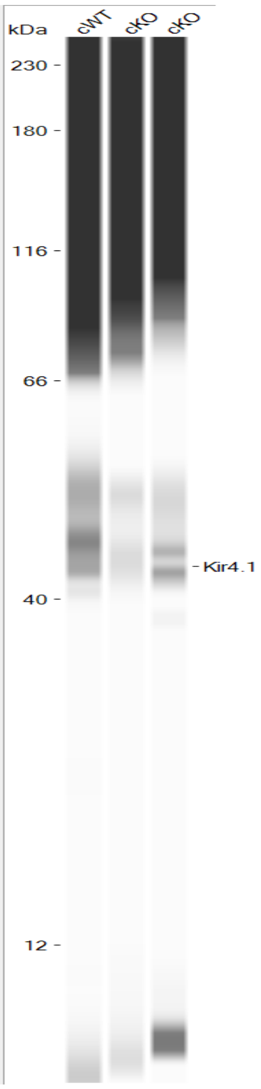

Panel L, N, P (loading control)

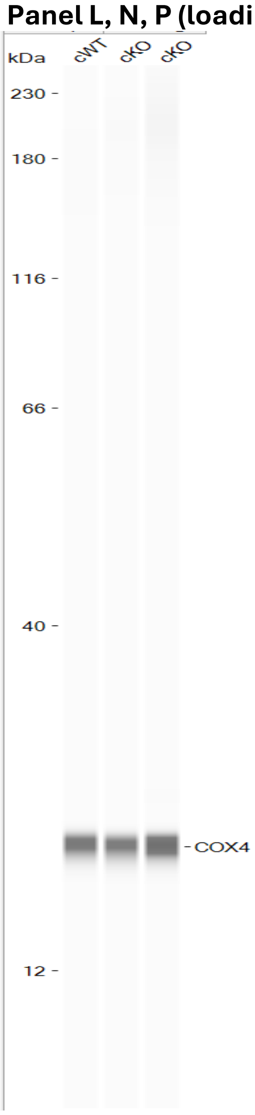

Source Data Figure 7 (Cont'd)

Panel L

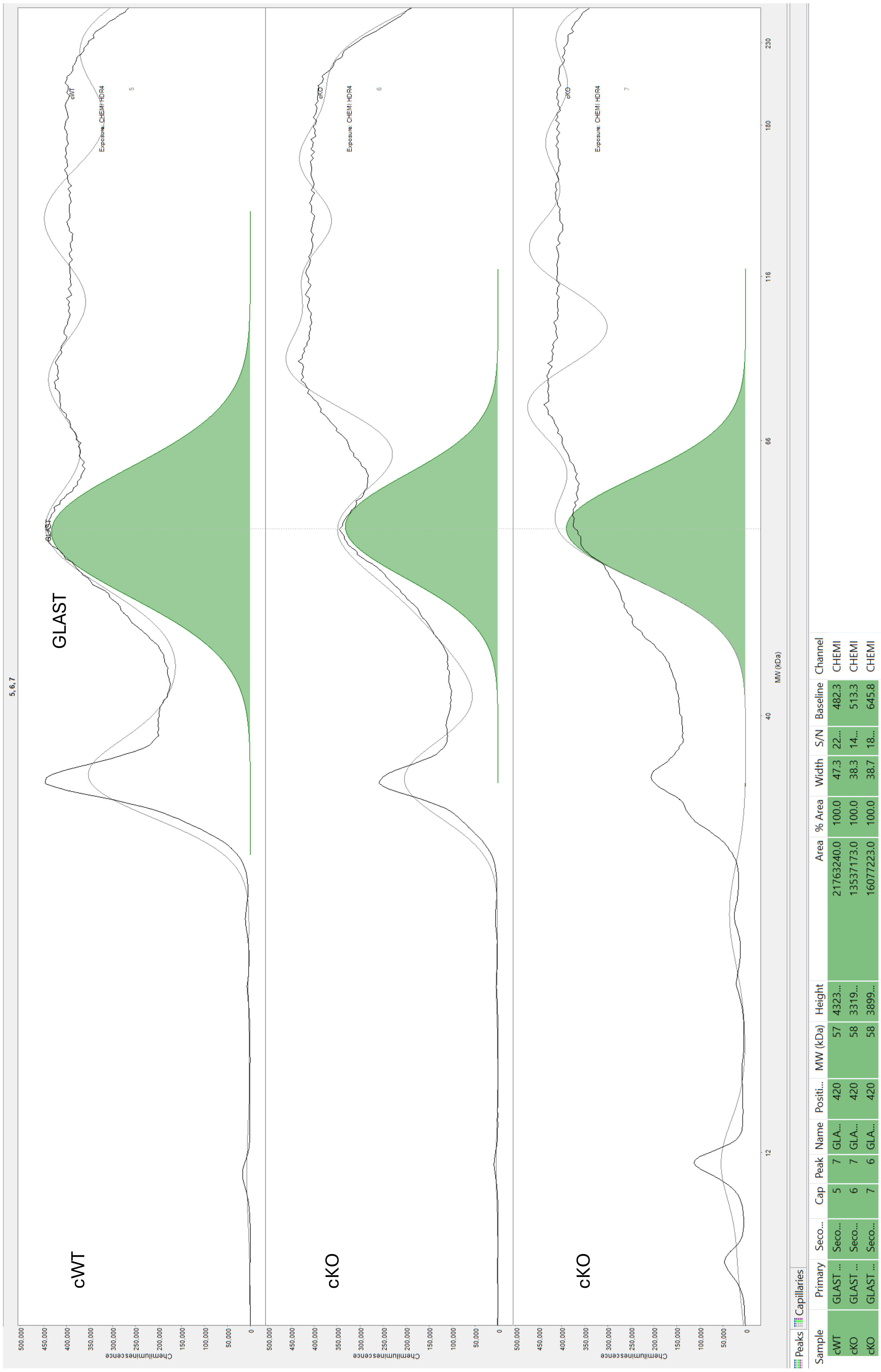

Sample	Primary	Seco...	Cap	Peak	Name	Positi...	MW (kDa)	Height	Area	% Area	Width	S/N	Baseline	Channel
cWT	GLAST ...	Seco...	5	7	GLA...	420	57	4323...	21763240.0	100.0	47.3	22...	482.3	CHEMI
cKO	GLAST ...	Seco...	6	7	GLA...	420	58	3319...	13537173.0	100.0	38.3	14...	513.3	CHEMI
cKO	GLAST ...	Seco...	7	6	GLA...	420	58	3899...	16077223.0	100.0	38.7	18...	645.8	CHEMI

Source Data Figure 7 (Cont'd)

Panel N

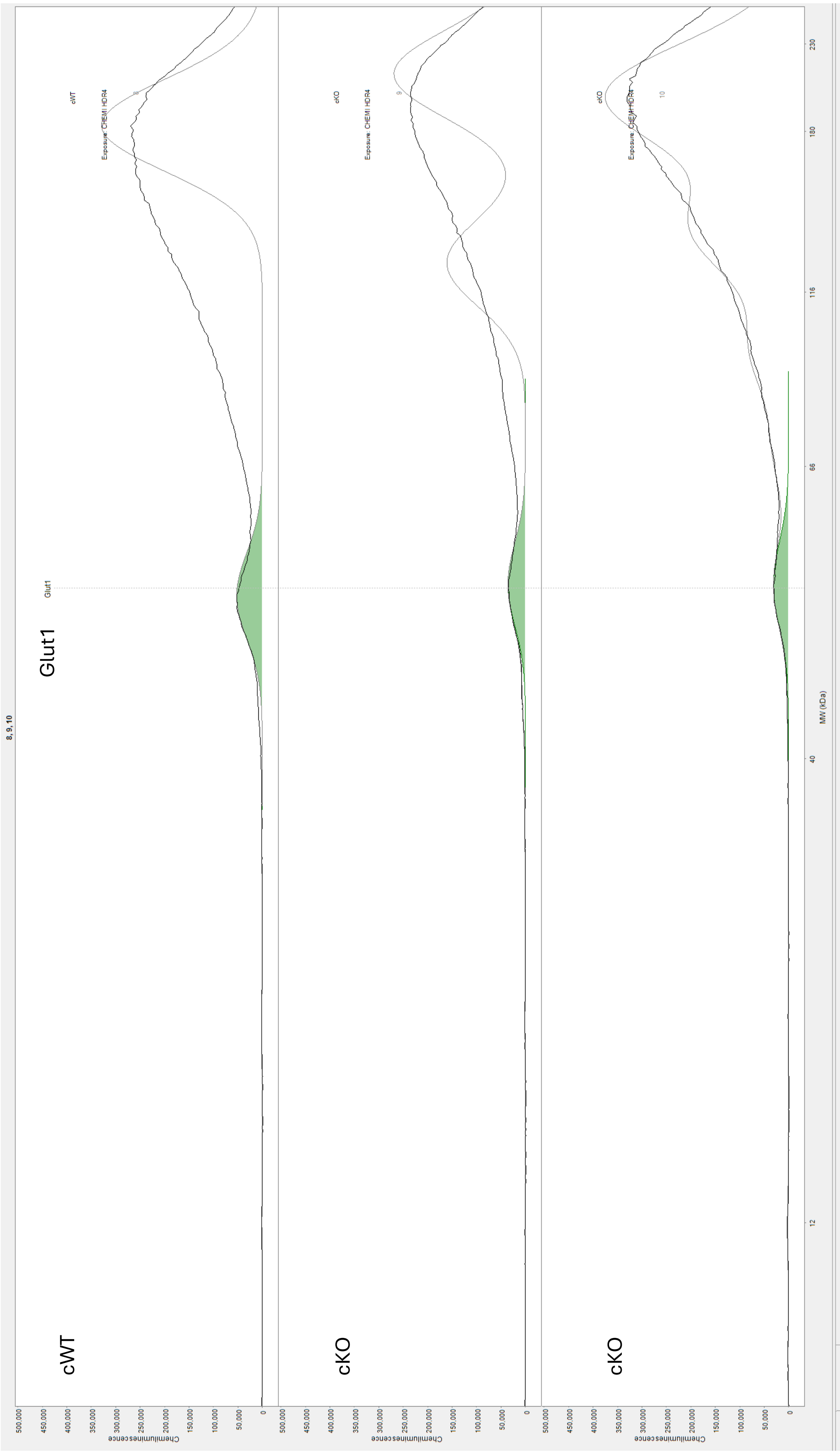

Peaks | Capillaries

Sample	Primary	Seco...	Cap	Peak	Name	Positi...	MW (kDa)	Height	Area	% Area	Width	S/N	Baseline	Channel
cWT	Glut1 Rb	Seco...	8	5	Glut1	410	55	5246...	1687195.3	100.0	30.2	59...	3345.4	CHEMI
cKO	Glut1 Rb	Seco...	9	5	Glut1	414	56	3588...	1094013.2	100.0	28.6	32...	3275.6	CHEMI
cKO	Glut1 Rb	Seco...	10	5	Glut1	412	55	3088...	990367.5	100.0	30.1	25...	4043.4	CHEMI

Source Data Figure 7 (Cont’d)

Panel P

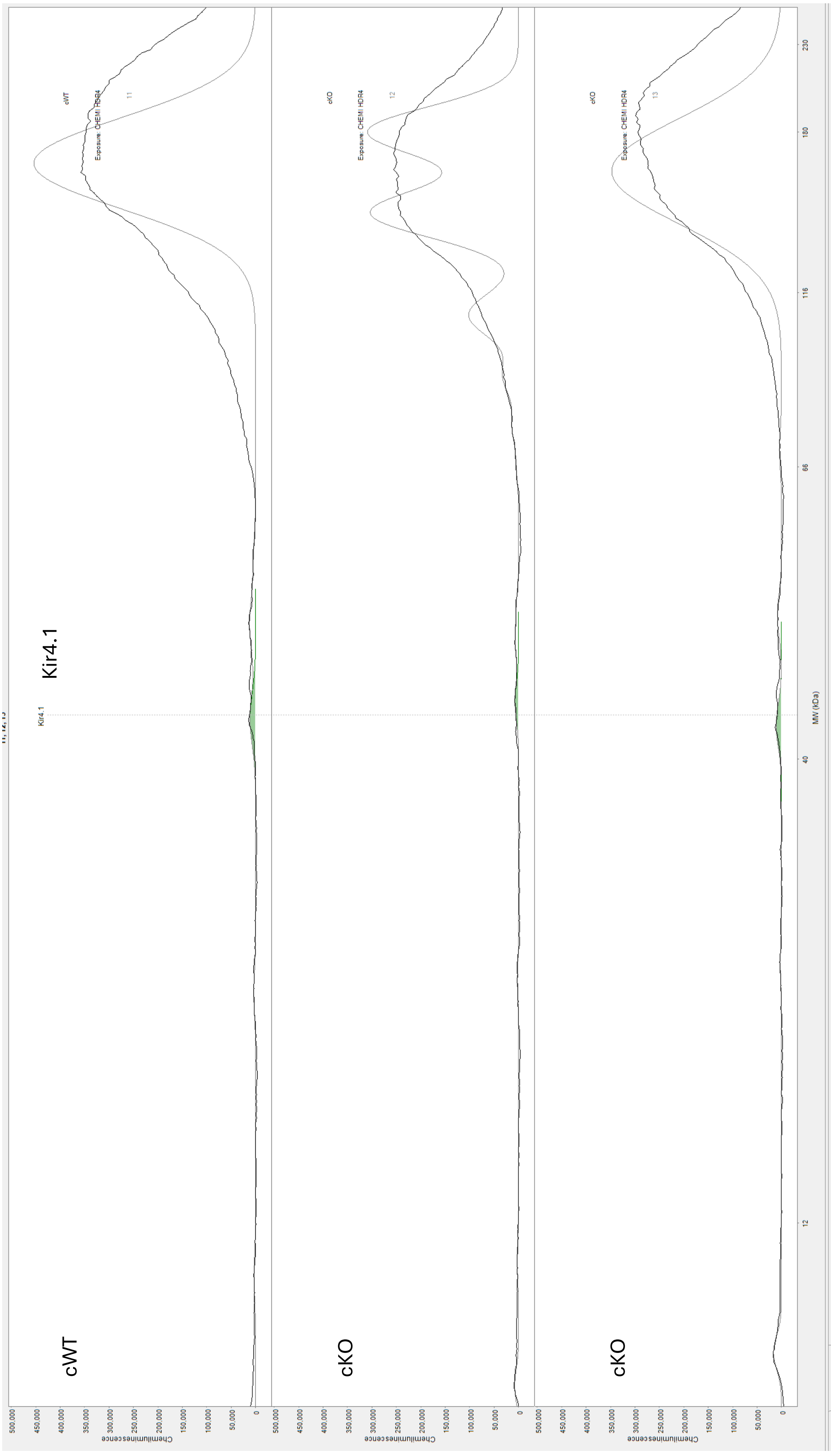

Sample	Primary	Seco...	Cap	Peak	Name	Positi...	MW (kDa)	Height	Area	% Area	Width	S/N	Baseline	Channel
cWT	Kir4.1 Rb	Seco...	11	6	Kir4.1	375	44	1138...	219130.1	100.0	18.1	26.6	27970.9	CHEMI
cKO	Kir4.1 Rb	Seco...	12	7	Kir4.1	387	45	7545.1	104613.6	100.0	13.0	12.1	25750.7	CHEMI
cKO	Kir4.1 Rb	Seco...	13	5	Kir4.1	372	43	1111...	179886.8	100.0	15.2	20.5	23400.0	CHEMI

Source Data Figure 7 (Cont'd)

N of 3&4

Panel L, N, P (loading control)

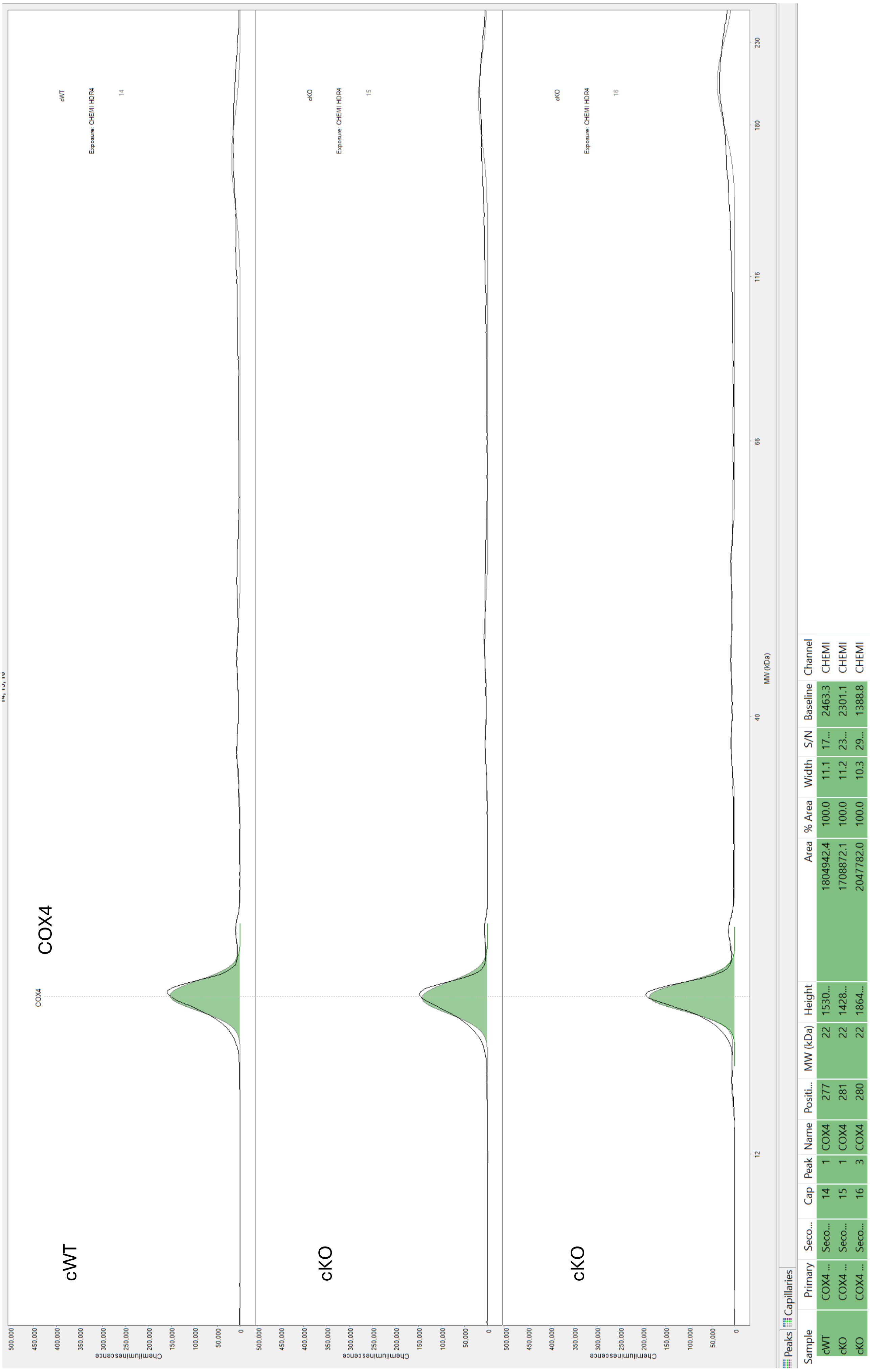

Sample	Primary	Seco...	Cap	Peak	Name	Positi...	MW (kDa)	Height	Area	% Area	Width	S/N	Baseline	Channel
cWT	COX4 ...	Seco...	14	1	COX4	277	22	1530...	1804942.4	100.0	11.1	17...	2463.3	CHEMI
cKO	COX4 ...	Seco...	15	1	COX4	281	22	1428...	1708872.1	100.0	11.2	23...	2301.1	CHEMI
cKO	COX4 ...	Seco...	16	3	COX4	280	22	1864...	2047782.0	100.0	10.3	29...	1388.8	CHEMI
